# The Transcriptomic Response of the Murine Thyroid Gland to Iodide Overload and the Role of the Nrf2 Antioxidant System

**DOI:** 10.3390/antiox9090884

**Published:** 2020-09-18

**Authors:** Dionysios V. Chartoumpekis, Panos G. Ziros, Ilias Georgakopoulos-Soares, Adam A. T. Smith, Ana Claudia Marques, Mark Ibberson, Peter A. Kopp, Ioannis Habeos, Ioannis P. Trougakos, Nicholas K. H. Khoo, Gerasimos P. Sykiotis

**Affiliations:** 1 Service of Endocrinology and Diabetology, Lausanne University Hospital, and Faculty of Biology and Medicine, University of Lausanne, 1011 Lausanne, Switzerland; dchart@upatras.gr (D.V.C.); panos.ziros@chuv.ch (P.G.Z.); peter.kopp@chuv.ch (P.A.K.); 2 Division of Endocrinology, Department of Internal Medicine, University of Patras, 26504 Patras, Greece; ihabeos@med.upatras.gr; 3 Department of Bioengineering and Therapeutic Sciences, University of California, San Francisco, San Francisco, CA 94158, USA; ilias.georgakopoulossoares@ucsf.edu; 4 Institute for Human Genetics, University of California, San Francisco, San Francisco, CA 94158, USA; 5 Department of Computational Biology, University of Lausanne, 1015 Lausanne, Switzerland; alexander.smith@baker.edu.au (A.A.T.S.); anaclaudia.marques@unil.ch (A.C.M.); 6 Vital-IT Group, Swiss Institute of Bioinformatics, 1015 Lausanne, Switzerland; mark.ibberson@sib.swiss; 7 Department of Cell Biology and Biophysics, Faculty of Biology, National and Kapodistrian University of Athens, 15784 Athens, Greece; itrougakos@biol.uoa.gr; 8 Department of Pharmacology & Chemical Biology, School of Medicine, University of Pittsburgh, Pittsburgh, PA 15261, USA; nkhoo@pitt.edu

**Keywords:** Kelch-like ECH-associated protein 1 (Keap1), Nfe2l2, RNA-Seq, iodine, oxidative stress, inflammation, fibrosis, immune response

## Abstract

Background: Thyroid follicular cells have physiologically high levels of reactive oxygen species because oxidation of iodide is essential for the iodination of thyroglobulin (Tg) during thyroid hormone synthesis. Thyroid follicles (the functional units of the thyroid) also utilize incompletely understood autoregulatory mechanisms to defend against exposure to excess iodide. To date, no transcriptomic studies have investigated these phenomena in vivo. Nuclear erythroid factor 2 like 2 (Nrf2 or Nfe2l2) is a transcription factor that regulates the expression of numerous antioxidant and other cytoprotective genes. We showed previously that the Nrf2 pathway regulates the antioxidant defense of follicular cells, as well as *Tg* transcription and Tg iodination. We, thus, hypothesized that Nrf2 might be involved in the transcriptional response to iodide overload. Methods: C57BL6/J wild-type (WT) or Nrf2 knockout (KO) male mice were administered regular water or water supplemented with 0.05% sodium iodide for seven days. RNA from their thyroids was prepared for next-generation RNA sequencing (RNA-Seq). Gene expression changes were assessed and pathway analyses were performed on the sets of differentially expressed genes. Results: Analysis of differentially expressed messenger RNAs (mRNAs) indicated that iodide overload upregulates inflammatory-, immune-, fibrosis- and oxidative stress-related pathways, including the Nrf2 pathway. Nrf2 KO mice showed a more pronounced inflammatory–autoimmune transcriptional response to iodide than WT mice. Compared to previously published datasets, the response patterns observed in WT mice had strong similarities with the patterns typical of Graves’ disease and papillary thyroid carcinoma (PTC). Long non-coding RNAs (lncRNAs) and microRNAs (miRNAs) also responded to iodide overload, with the latter targeting mRNAs that participate mainly in inflammation pathways. Conclusions: Iodide overload induces the Nrf2 cytoprotective response and upregulates inflammatory, immune, and fibrosis pathways similar to autoimmune hyperthyroidism (Graves’ disease) and PTC.

## 1. Introduction

The thyroid gland normally produces high amounts of hydrogen peroxide (H_2_O_2_), which is necessary for the oxidation of iodine to iodide, the iodination of thyroglobulin (Tg), and ultimately the production of thyroid hormones, which are essentially iodinated derivatives of selected tyrosines of Tg. To maintain the reactive oxygen species at bay and prevent them from causing oxidative stress, thyroid cells employ a system of antioxidant enzymes such as glutathione peroxidase 2 (Gpx2) and thioredoxin reductase 1 (Txnrd1); these are induced further under conditions of excess iodide [1,2] secondary to the increase in reactive oxygen species [3]. We recently showed that the transcription factor nuclear erythroid factor 2 like 2 (Nfe2l2 or Nrf2) plays an important role in the positive regulation of these enzymes and is also a direct positive regulator of *Tg* transcription and a negative regulator of *Tg* iodination [2].

Nrf2 is central for the regulation of cytoprotective and antioxidant responses [4]. Kelch-like ECH associated protein 1 (Keap1) is a cytoplasmic protein that binds Nrf2 and targets it for proteasomal degradation. Keap1 acts as a redox sensor [5] through the oxidation of the sulfhydryl groups of specific cysteines which results in conformational changes in Keap1 that render it incapable of binding new Nrf2 molecules [6]. Nrf2 accumulates and enters the nucleus and induces its target genes via specific binding sequences called antioxidant response elements (AREs). In the last few years, a role of Nrf2 in thyroid physiology and pathophysiology has emerged [7]; Nrf2 upregulates *Tg* transcription [2] through AREs in a *Tg* enhancer [2,8], downregulates Tg iodination [2], and is activated in response to iodide exposure [2,9], as well as in thyroid carcinomas [10,11,12]. The Nrf2 pathway is of high medical interest due to its druggable nature, and Nrf2 inducers have been tested in clinical trials for cancer chemoprevention, chronic kidney disease, neurodegenerative diseases, and diabetes [13]. Natural compounds such as sulforaphane from broccoli sprout extracts can enhance Nrf2 signaling in thyroid follicular cells [2,14] and provide a promising method to enhance cytoprotection in the thyroid.

Thyroid follicles (the functional units of the thyroid) possess autoregulatory mechanisms to defend against exposure to excess iodide. Although the mechanisms underlying thyroid autoregulation have been in part elucidated from a physiological and biochemical perspective, their molecular and cell-biological basis remains incompletely understood. In particular, little is known about the transcriptomic signature associated with iodide excess. Iodide exposure was shown to increase oxidative stress in the thyroid, as assessed by protein carbonylation [2,15], and to increase the levels of reactive oxygen species (ROS) as measured indirectly by DCF (2′,7′-dichlorofluorescein) fluorescence in cell culture [1,16]. However, no in vivo studies have assessed the transcriptomic response of the thyroid to iodide overload to identify pathways that can explain or respond to this iodide-induced increase in oxidative stress. Some microarray-based studies were published with relevance to the effect of iodide excess in cultured human thyroid follicles from Graves’ disease patients [17,18,19] and in the rat FRTL-5 thyroid cell line [20]. Other studies employed serial analysis of gene expression (SAGE) in iodide-treated rat thyroid follicular PCCl3 cells [1] or real-time PCR-based gene expression analysis in iodide-exposed mouse endoderm cells differentiating into thyrocytes [21]. In the present study, we performed, to our knowledge, the first in vivo transcriptomic analysis of the response of thyroid to excess iodide in wild-type (WT) and Nrf2 knockout (KO) mice to determine the role of the Nrf2 pathway in this model.

## 2. Materials and Methods

### 2.1. Mice

C57BL/6J *Nrf2+/−* mice, obtained from RIKEN BRC (Tsukuba, Japan) [22], were used to generate wild-type (WT) and Nrf2 knockout mice (KO). For iodide challenge, male WT and KO mice (three to four months old) fed a standard diet (product number 4RF21, Mucedola, Italy, containing 1 mg/kg iodine) were supplied with normal tap water with or without 0.05% sodium iodide (NaI) (Sigma, St Louis, MO, USA) for seven days. Thus, the study comprises the following four groups of mice: WT mice on regular tap water (WT control (WTC), *n* = 9), WT mice on 0.05% NaI (WT on iodide (WTI), *n* = 12), KO mice on regular tap water (KO control, (KOC), *n* = 10), and KO mice on 0.05% NaI (KO on iodide (KOI), *n* = 10). Mice were housed in the animal facility of the University of Patras Medical School in temperature-, light-, and humidity-controlled rooms with a 12 h light/dark cycle. All animal procedures were approved by the institutional review board of the University of Patras Medical School (approval ID 15/2008) and were in accordance with EC Directive 86/609/EEC.

### 2.2. Next-Generation Messenger RNA (mRNA) Sequencing

RNA was prepared from thyroids as previously described so as to preserve the microRNA (miRNA) fraction [23]. WTC (*n* = 3), WTI (*n* = 4), KOC (*n* = 4), and KOI (*n* = 5) thyroid RNA samples were submitted to Exiqon (Aarhus, Denmark) for RNA quality assessment, library preparation, RNA sequencing (RNA-Seq), and initial bioinformatic analysis. RNA-Seq was performed on a NextSeq 500 instrument (Illumina Inc., San Diego, CA, USA) in three runs with an estimated 69.5 million reads/sample with a read length of 1 × 50 bp. Raw sequencing data were uploaded to Zenodo (https://doi.org/10.5281/zenodo.4005813) with open-access rights, as well as to National Center for Biotechnology Information (NCBI) Gene Expression Omnibus (series GSE157121). Alignment and mapping were performed using the TopHat and Cufflinks suites [24]. The reference genome used was *Mus musculus* GRCm38, and on average 97.6% of reads from each sample mapped to this genome. For mRNAs, the iDEP platform [25] was used to pipeline the gene expression analysis. To eliminate genes with low expression, minimum CPM (counts per million reads) was set to 1 for all samples (Figure S1A, Supplementary Materials); edgeR [26] was used to transform count data for clustering and PCA analysis. DESeq2 [27] was used to analyze the differential expression of genes with a minimum fold change (increase or decrease) of 1.5 and with a false discovery rate (FDR) cutoff set at <0.05. For long non-coding RNAs (lncRNAs), the HTSeq framework was used to count the read hits [28] and DESeq2 was used for differential gene expression analysis.

### 2.3. Next-Generation miRNA Sequencing

The same samples used for mRNA sequencing were also used for miRNA sequencing by Exiqon on a NextSeq 500 instrument with an average of 18 million reads per sample and with a 51 bp single-end read length. Raw sequencing data were uploaded to Zenodo (https://doi.org/10.5281/zenodo.4005813) with open-access rights. Sequence reads were processed to remove the adaptor sequences and reformatted to FASTA files using the FASTX-Toolkit. To generate count data, the raw sequences were compared to mature mouse miRNA sequences (from miRBase Version 21) and other non-coding RNA sequences (RNACentral Version 5) using MEGABLAST with a word size of eight nucleotides. The criteria for counting a sequence match were as follows: %query ≥90% of the target sequence and ≤2 mismatches over the alignment. The matches against miRBase were parsed and the top matches were selected. To avoid ambiguous mappings, if a sequence had >1 top match against different database sequences, it was excluded from subsequent analyses. Matches to RNACentral were only taken into account for sequences not matching to miRBase. Next, the data were filtered using R (R Foundation for Statistical Computing, Vienna, Austria) by removing miRNAs that (i) did not have a perfect match to ≥90% of the length of the target sequence, and (ii) showed low overall expression; the counts were first converted to CPM, and miRNAs that did not have ≥10 CPM in ≥3 samples were filtered out. The counts were then normalized using edgeR’s TMM (trimmed mean of M values) method to correct for differences in the number of reads mapping to miRNAs. To calculate the fold expression changes, differential expression of miRNAs was called using edgeR with a nominal *p*-value cutoff of 0.05 and a minimal absolute log_2_ fold change cutoff of 0.10.

### 2.4. Pathway Analysis

The mRNAs that were differentially expressed for the comparisons shown in relevant figures were uploaded to the Ingenuity Pathway Analysis (IPA) platform (Qiagen, Redwood City, CA, USA), and a “core analysis” was run to extract the enriched canonical pathways and the upstream regulators of the differentially expressed mRNAs. The microRNA Target Filter of IPA was employed to match the differentially expressed miRNAs upon iodide exposure with their known or predicted mRNA targets that were also found to be differentially expressed. IPA match analysis was used to compare our datasets with other publicly available datasets curated by IPA.

### 2.5. Real-Time PCR

Complementary DNA (cDNA) was synthesized from all the mice in the study (not just from the thyroids used for next-generation sequencing (NGS) using 250 ng of thyroid RNA and the SuperScript VILO cDNA Synthesis Kit (Invitrogen). Real-time PCRs were performed on StepOnePlus or ViiA 7 Real-Time PCR System instruments (Applied Biosystems, Foster City, CA, USA) using FAST SYBR green (Kapa Biosystems, Woburn, MA, USA) under the following conditions: 3 min at 95 °C, followed by 40 cycles of 10 s at 95 °C and 25 s at 60 °C. Gene expression changes were calculated on the basis of the ΔΔ*Ct* method. Standard curves were used to assess the efficiency of each reaction, and melt curve analysis was included to verify that each PCR reaction had a single product. Genes related with thyroid economy and oxidative stress response were selected randomly for gene expression analysis by real-time PCR. The primer sequences were previously reported [2].

### 2.6. Statistics

For calling differentially expressed genes (DEGs), the statistical testing methods employed were those embedded in the aforementioned suites (DESeq2 for mRNA and lncRNA data, and edgeR for miRNA data). For other analyses, specific details are provided in the respective figure legends. Statistical significance was set at *p* < 0.05 for all analyses.

## 3. Results

### 3.1. Iodide Exposure of WT and Nrf2 KO Mice Leads to Distinct Gene Expression Clusters Enriched for Immune, Inflammatory, and Antioxidant Response Functions

**Figure 1:**
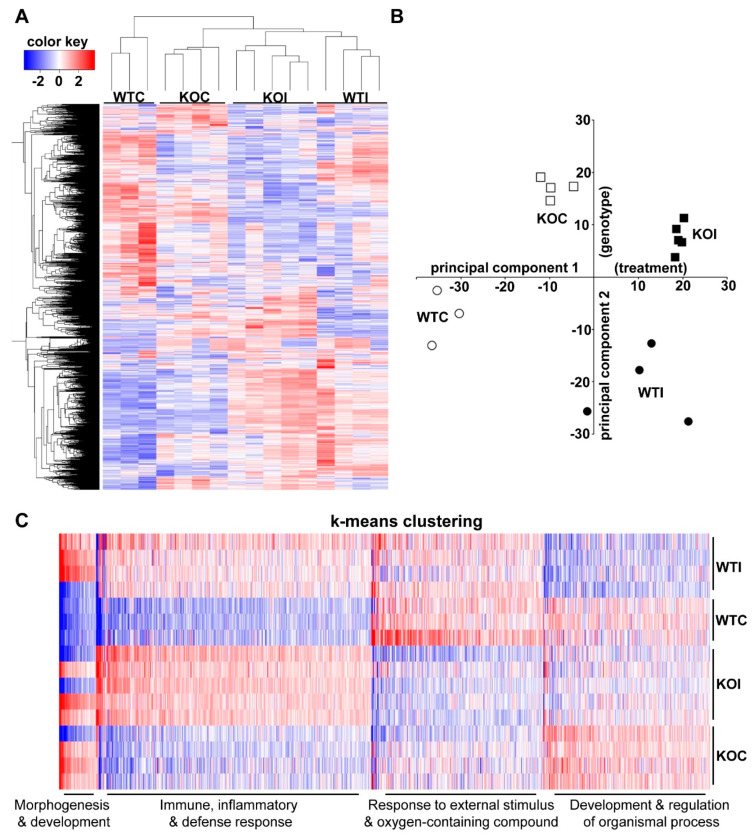
(**A**) Heatmap and clustering of the 1500 genes showing the most variable expression in the thyroids of the various cohorts of mice used for RNA sequencing (RNA-Seq). The heatmap was clustered by Euclidean distance and average linkage. Red color in the heatmap indicates higher expression and blue color indicates lower expression. (**B**) Principal component analysis (PCA) of RNA-Seq data. Of the total variance in gene expression, 29% can be attributed to PC1, which is correlated with treatment (*p* = 1.14 × 10^−4^), and 16% can be attributed to PC2, which is correlated with genotype (*p* = 3.74 × 10^−5^). (**C**) The 2000 most variable genes were clustered into groups using k-means clustering on the basis of their expression pattern across all samples followed by enrichment analysis for each cluster. The number of clusters was set to 4 following the “elbow method” (Figure S1B, Supplementary Materials). The enriched pathways for each cluster along with the *p*-values and genes in each pathway are shown in Table S1 (Supplementary Materials). Representative pathways for the largest clusters are indicated in the figure. Red color in the heatmap indicates higher expression while blue indicates lower expression. WTC: wild-type control mice (regular water); WTI: iodide-treated wild-type mice (0.05% NaI in water); KOC: nuclear erythroid factor 2 like 2 (Nrf2) knockout mice (regular water); KOI: iodide-treated Nrf2 knockout mice (0.05% NaI in water).

Across the whole cohort of mice used for RNA-Seq, the genes showing the most variable thyroidal expression among the mice showed consistent profiles within each group (Figure 1A). This means that the effects of genotype (WT or KO), iodide treatment, or their combination each led to a distinct gene expression signature. This is also evident from the principal component analysis (PCA) of gene expression values (Figure 1B), where the samples clustered clearly depending on treatment (principal component 1-PC1) and genotype (principal component 2-PC2). Performing k-means clustering of the genes with the most variable expression showed that the major pathways enriched for each main cluster referred mainly to immune, inflammatory and defense response, and response to external stimulus and oxygen-containing compounds. Immune, inflammatory, and defense responses tended to be upregulated with iodide treatment (illustrated in the heatmap by the predominance of blue color in samples from control mice and the predominance of red color in samples from iodide-exposed mice) (Figure 1C). Responses to external stimulus and oxygen-containing compound tended to be downregulated in KO samples (Figure 1C). Table S1 (Supplementary Materials) lists all enriched pathways.

### 3.2. Iodide Excess Leads to Differential Gene Expression in the Thyroid of WT and Nrf2 KO Mice, in Particular in Cytoprotective/Antioxidant and Thyroid Economy-Related Genes

**Figure 2:**
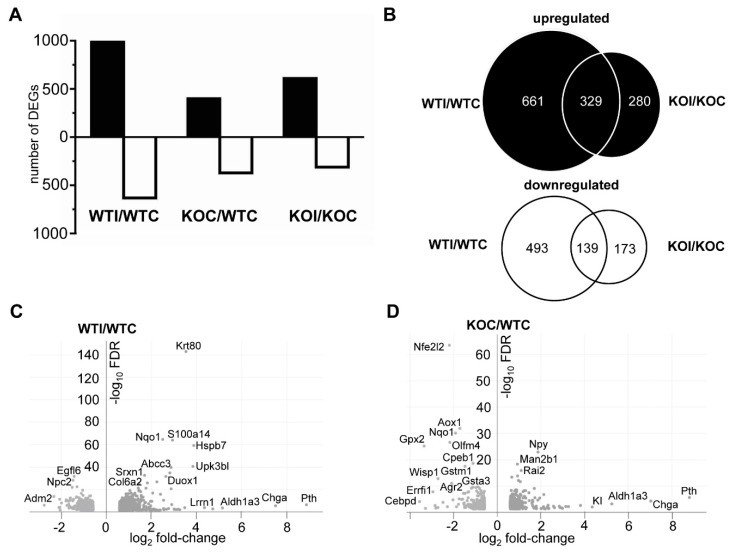
(**A**) Number of differentially expressed genes (DEGs) (messenger RNAs (mRNAs)) per comparison. The effect of iodide on wild-type (WT) mice (WTI/WTC) and on Nrf2 knockout (KO) mice (KOI/KO), as well as the effect of genotype (KOC/WTC) on gene expression, were assessed by the DESeq2 method, with a false discovery rate (FDR) set at 0.05 and the minimum gene expression fold change set at 1.5. Table S2 (Supplementary Materials) contains full lists of DEGs. (**B**) Venn diagrams with upregulated and downregulated genes (mRNAs) after iodide in WT and Nrf2 KO mice. (**C**) Volcano plots of genes that are differentially expressed (FDR < 0.05, fold change ≥ 1.5) in WT mice after iodide (WTI/WTC). (**D**) Volcano plots of genes that are differentially expressed (FDR < 0.05, fold change ≥ 1.5) in Nrf2 KO mice versus WT mice (KOC/WTC) without excess iodide. Some gene names are indicatively noted.

**Figure 3:**
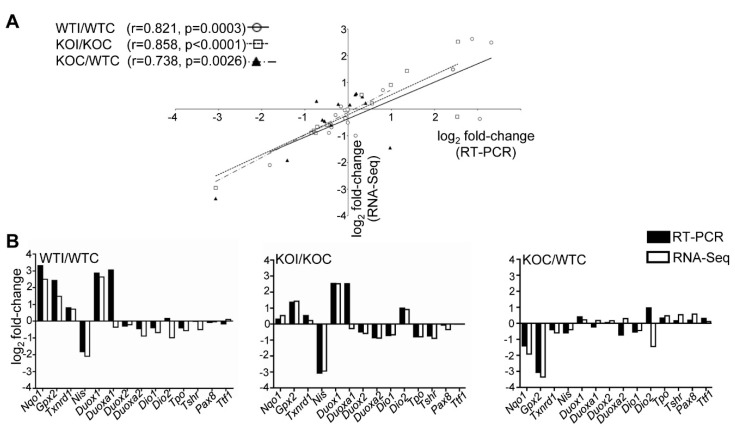
(**A**) RNA-Seq data validation by RT-PCR. Correlation of the expression fold changes between RNA-Seq and RT-PCR of 14 selected genes in WT mice after iodide treatment (WTI/WTC, circles and solid line, Pearson’s correlation coefficient *r* = 0.821, *p* = 0.0003), in Nrf2 KO mice after iodide (KOI/KOC, squares and dashed line, Pearson’s *r* = 0.858, *p* < 0.0001), and in Nrf2 KO versus WT without excess iodide (KOC/WTC, triangles and dashed line, Pearson’s *r* = 0.738, *p* = 0.0026). (**B**) Fold changes of each of the genes used for correlation analysis depicted according to the measurement method (black bars, RT-PCR; white bars, RNA-Seq) and the relevant comparison (WTI/WTC, KOI/KOC, or KOC/WTC). RT-PCR was performed in all study samples (WTC, *n* = 9; WTI, *n* = 12; KOC, *n* = 10; KOI, *n* = 10), while RNA-Seq was performed in 3/9, 4/12, 4/10, and 5/10 samples, respectively.

Iodide increased the thyroidal expression of 990 genes (mRNAs) in WT mice and 609 genes in Nrf2 KO mice by ≥50%, with 329 of them being common between the two genotypes. Fewer genes were downregulated: 632 in WT and 609 in KO mice, with 312 in common (Figure 2A,B). The volcano plot in Figure 2C depicts the DEGs in response to iodide in WT mice. The Nrf2-regulated genes *Nqo1* and *Srxn1* [29,30] were upregulated by iodide, as was the thyroid-specific gene *Duox1* that is important in thyroid hormone synthesis as it generates reactive oxygen species. Other genes upregulated by iodide included the cellular-stress response chaperone *Hspb7* along with the cytoskeletal-fibrosis genes keratin 80 (*Krt80*) and collagen 6 (*Col6a2*). In mice not treated with iodide, deletion of *Nrf2* also affected gene expression, with 371 downregulated and 399 upregulated genes in KO mice (Figure 2A,B). Genes related to cytoprotection and antioxidant response (e.g., *Gsta3*, *Gstm1*, *Nqo1*, *Gpx2*), as well as the H_2_O_2_-producing gene *Aox1* that is usually decreased in thyroid cancers [31], were all downregulated in thyroids of KO mice (Figure 2D), as found previously in other tissues [32,33]. Full lists of DEGs are provided in Table S2 (Supplementary Materials). Genes that were downregulated in KO mice and, thus, constitute potential Nrf2 target genes are listed in Table S3 (Supplementary Materials), along with compatible expression changes in other tissues from public databases. To verify gene expression changes that emanate from the thyroid samples that underwent RNA-Seq, the relative expression of three antioxidant-cytoprotective genes (*Nqo1*, *Gpx2, Txnrd1*) and 11 genes important for thyroid physiology (*Nis, Duox1, Duoxa1, Duox2, Duoxa2, Dio1, Dio2, Tpo, Tshr, Pax8*, and *Ttf1*) were assessed in all study samples (*n* = 41) by RT-PCR (Figure 3A,B). For each of the three comparisons (WTI/WTC, KOI/KOC, KOC/WTC), there was a highly significant correlation (*r* > 0.73) between the fold changes obtained from RNA-Seq (*n* = 3–5 samples per group) and those obtained from RT-PCR (*n* = 9–12 samples per group) (Figure 3A). This confirmed the results of RNA-Seq from both a technical and a biological perspective.

### 3.3. Nrf2 Mediates the Antioxidant Response and Ameliorates the Inflammatory–Immune–Fibrosis Response to Excess Iodide

**Figure 4:**
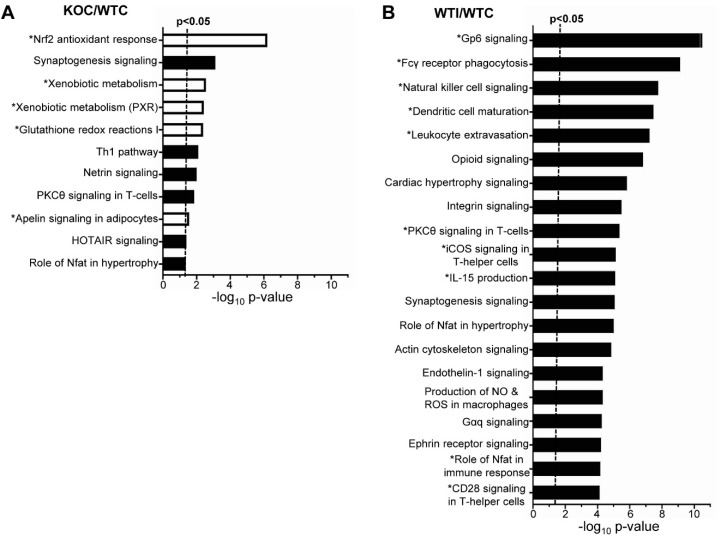
(**A**) Top canonical pathways enriched among DEGs (mRNAs) in Nrf2 KO mice compared to WT mice (KOC/WTC) ranked by *p*-value, as assessed by Ingenuity Pathway Analysis (IPA). The absolute *z*-score was set at ≥1.5. White bars indicate downregulation of the respective pathway (negative *z*-score), while black bars indicate upregulation of the respective pathway (positive *z*-score). The asterisk (*) before the pathway name indicates that it is relevant to Nrf2 signaling. (**B**) Top canonical pathways enriched in WT mice after iodide (WTI/WTC) ranked by *p*-value (as assessed by IPA). Absolute *z*-score was set at ≥2. Black bars indicate upregulation of the respective pathway (positive *z*-score). The asterisk (*) before the pathway name indicates that it is relevant to inflammatory/immune processes. Only pathways with −log_10_ *p*-value > 4 are depicted. Table S3 (Supplementary Materials) lists all enriched pathways with absolute *z*-score ≥ 2 and *p* < 0.05.

**Figure 5:**
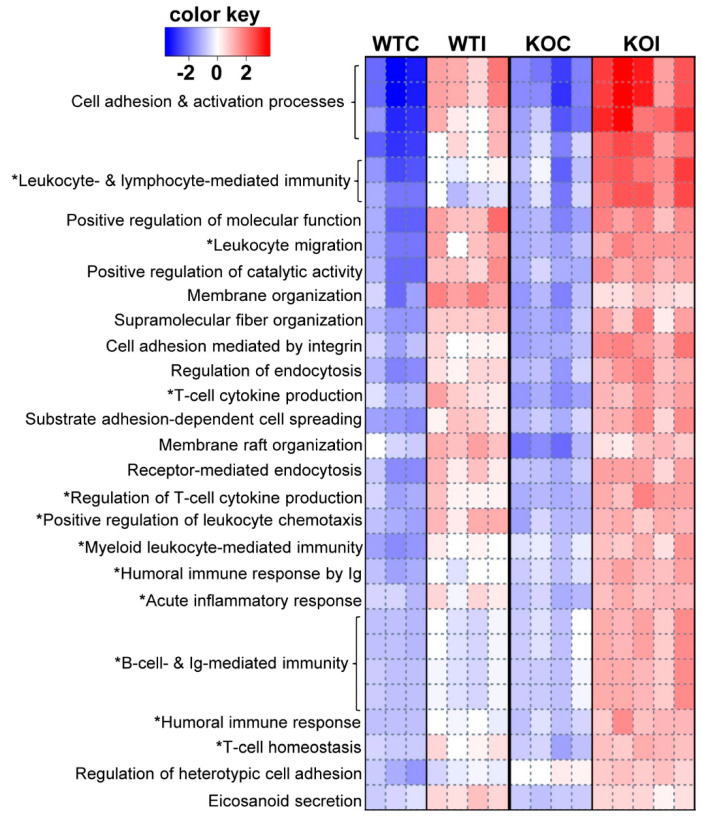
Pathway analysis of genes (mRNAs) that respond differentially to iodide among Nrf2 KO and WT mice using Parametric Gene Set Enrichment Analysis (PGSEA). The heatmap depicts the trend for each Gene Ontology (GO) biological process for each sample. Red squares indicate upregulation (positive *z*-score), while blue squares indicate downregulation (negative *z*-score). GO terms marked with an asterisk (*) refer to processes relevant to inflammation/autoimmunity. The top 30 pathways are shown, with FDR set at <0.05. Table S5 (Supplementary Materials) shows the *z*-score values for each pathway per sample and the respective *p*-values for each pathway.

**Figure 6:**
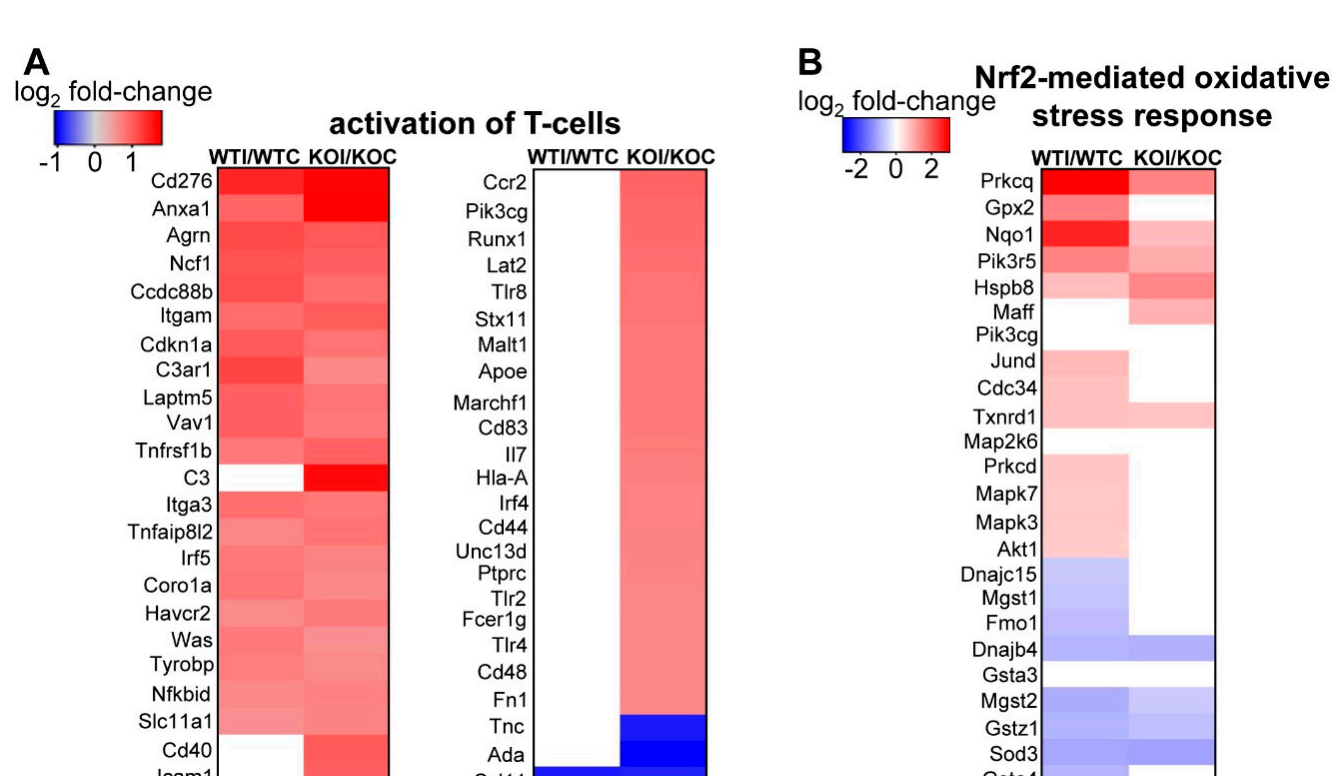
(**A**) Heatmap of genes (mRNAs) that participate in the “activation of T-lymphocytes” process, which is enriched in Nrf2 KO mice after iodide as compared to WT mice after iodide. This process is upregulated in Nrf2 KO mice after iodide (*z*-score = 3.466, IPA algorithm), while, in WT mice, it is not enriched at all after iodide. Red color indicates upregulation by ≥1.5-fold, while blue color indicates downregulation by ≥1.5-fold (*p* < 0.05), and white color indicates gene expression change <1.5-fold and/or *p* ≥ 0.05. (**B**) Heatmap of genes (mRNAs) that participate in the “Nrf2-mediated oxidative stress response”, which is enriched in Nrf2 KO mice treated after iodide as compared to WT mice after iodide. This pathway is upregulated in WT mice after iodide (*z*-score = 2.333, IPA algorithm), while, in Nrf2 KO mice, it is not enriched at all after iodide. Color-coding in the heatmap is similar to that in panel A.

**Figure 7:**
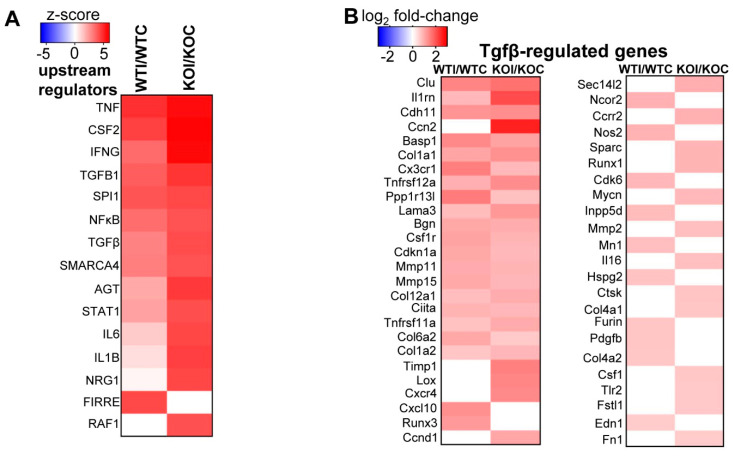
(**A**) Comparison of predicted upstream regulators of mRNAs differentially expressed after iodide treatment in WT mice as compared to Nrf2 KO mice (WTI/WTC vs. KOI/KOC, *z*-score ≥4 and *p* < 0.01). Red color indicates upregulation (positive *z*-score), while white color indicates absence of enrichment of the upstream regulator. The graph was generated by the comparison analysis of IPA. (**B**) Gene expression heatmap of genes that participate in the fibrosis pathway, which is predicted to be upregulated by the enriched upstream regulator TGFβ after iodide. Red color indicates ≥1.5-fold upregulation (*p* < 0.05), while white color indicates gene expression change <1.5-fold and/or *p* ≥ 0.05. The right column continues after the left one. Genes are presented from top to bottom from lower to higher *p*-value in either comparison (WTI/WTC, KOI/KOC).

As expected, under control conditions, Nrf2 KO mice showed downregulation of Nrf2-related pathways in their thyroids (Nrf2-mediated oxidative stress response, xenobiotic metabolism, glutathione redox reactions) (marked by an asterisk in Figure 4A). On the other hand, some pathways with relevance to inflammatory/immune response were upregulated in KO mice (Th1 pathway, PKCθ signaling in T cells) (Figure 4A). In WT mice, several inflammatory and immune response pathways were upregulated after iodide exposure (marked by an asterisk in Figure 4B). In addition, pathways related to production of ROS and the Nrf2-mediated oxidative stress response were also upregulated (Figure 4B and Table S4, Supplementary Materials). Although enriched upregulated pathways in KO mice after iodide appear to mostly overlap with those in WT mice, the KO mice tend to show a more potent immune/inflammatory response (darker red color, marked by an asterisk in Figure 5; full list in Table S5, Supplementary Materials). Such processes include the acute inflammatory response, leukocyte- and lymphocyte-mediated immunity, leukocyte migration, humoral immune response, and T-cell homeostasis. As shown in Figure 5, all these processes were significantly upregulated in KO mice after iodide compared to WT mice (red squares vs. white or blue squares in the heatmap). An example of such a pathway that is relevant to thyroid immunity (“activation of T lymphocytes”) and is more potently upregulated in KO mice is shown in Figure 6A as a heatmap of genes that participate in this pathway and are mostly upregulated after iodide. For control purposes, and to highlight the activation of Nrf2 signaling upon iodide exposure, Figure 6B shows that the Nrf2 pathway was upregulated after iodide in WT mice but not in Nrf2 KO mice, as expected; most of the known cytoprotective/antioxidant genes were upregulated in WT mice but not in Nrf2 KO mice. Inflammatory cytokines (Tumor necrosis factor- TNF, Colony stimulating factor 2 -CSF2, Interferon gamma- IFNG, Interleukin 6 -IL-6), Nuclear factor kappa B- NF-κB signaling, and Transforming growth factor beta- TGFβ signaling were among the highly enriched upstream regulators of this iodide-induced transcriptional response, highlighting the involvement of inflammatory and fibrosis pathways (Figure 7A). The upstream regulators of these pathways showed higher *z*-scores in Nrf2 KO mice after iodide, which is another indication that the inflammation–immune and fibrosis pathways were more potently upregulated in the *Nrf2*-disrupted mice. For instance, TGFβ showed a higher *z*-score as an upstream regulator of its target fibrosis genes in Nrf2 KO mice after iodide exposure than in WT mice (*z*-score = 4.171 vs. 2.956, respectively). Expression of TGFβ target genes was mostly increased after iodide exposure in both genotypes, but overall to a larger degree in Nrf2 KO mice (Figure 7B).

### 3.4. Iodide Excess Affects the Expression of lncRNAs and miRNAs in Thyroid

**Figure 8:**
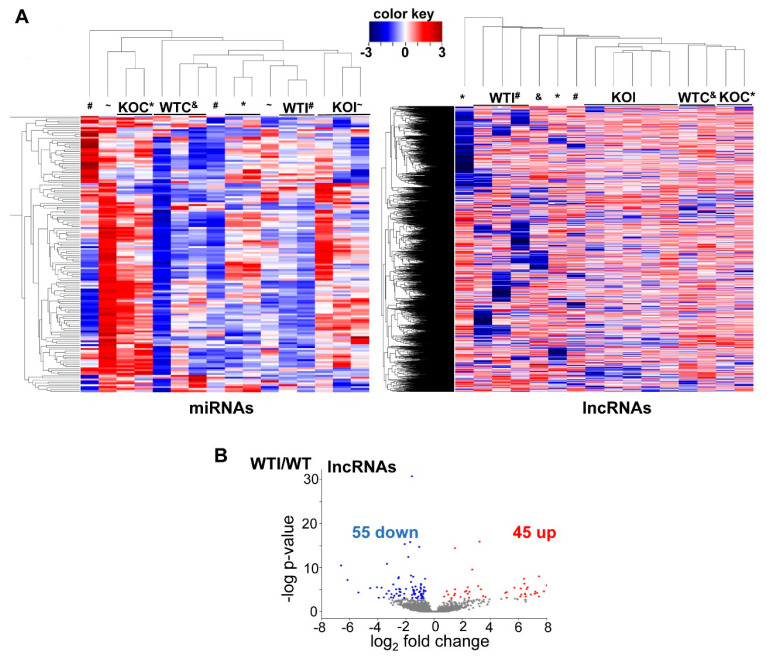
(**A**) Heatmap and clustering of differentially expressed microRNAs (miRNAs) and long non-coding RNAs (lncRNAs) based on standardized expression values. Samples are in columns and lncRNAs or miRNAs are in rows. Red color in the heatmap indicates higher expression and blue color indicates lower expression. Symbols indicate the group to which each sample belongs: &, WTC; #, WTI; *, KOC; ~, KOI. (**B**) Volcano plot of lncRNAs that were differentially expressed after iodide (WTI/WTC). Red color indicates significant upregulation, while blue color indicates significant downregulation (≥2-fold, *p* < 0.05), and gray color indicates gene expression change < 2-fold and/or *p* ≥ 0.05.

Even though knowledge on the involvement of small RNA species in the pathophysiology of thyroid, as well as in thyroid cancer, is increasing, there is no information in the bibliography regarding whether these RNA species in thyroid respond to iodide excess. To address this question, the expression of lncRNAs and miRNAs was analyzed in WT and Nrf2 KO mice with or without exposure to excess iodide. In contrast to the tight clustering patterns observed for mRNAs, neither miRNAs nor lncRNAs clustered ideally (Figure 8A); this was partly expected, because these species are both fewer in number and expressed at lower levels. One hundred lncRNAs responded to iodide, of which 45 were upregulated and 55 were downregulated (Figure 8B). Interestingly, the vast majority of the differentially expressed lncRNAs have not been previously characterized and, thus, further research is warranted to verify these results and identify their potential physiological role. Table S6 (Supplementary Materials) lists 47 differentially expressed lncRNAs that show detectable baseline expression in all samples and ≥2-fold change, and provides their exact genomic coordinates along with the respective neighboring genes.

Seventy miRNAs were found to respond to iodide, of which 44 were upregulated and 26 were downregulated (Tables S7 and S8, Supplementary Materials). As the sequencing for both mRNAs and miRNAs was performed in the exact same samples, we used the IPA microRNA Target Filter to pair the miRNAs upregulated or downregulated after iodide with their potential mRNA targets that were significantly downregulated or upregulated, respectively. Table S10 shows all 542 miRNA–mRNA pairings emanating from our dataset that were either experimentally observed in the past or predicted by relevant algorithms. Table S9 depicts the pairings of six miRNAs with their respective mRNA targets, which were experimentally verified in the bibliography curated by IPA.

### 3.5. Iodide Treatment Affects the Expression of miRNAs That Target mRNAs Which Are Part of the Observed Upregulated Inflammatory–Immune Response Pathways

**Figure 9:**
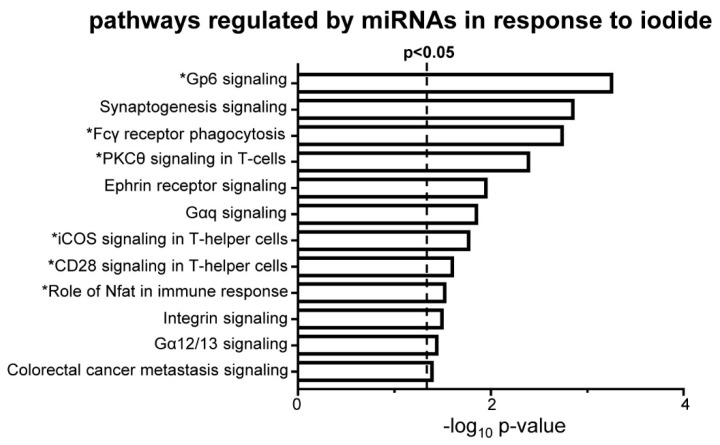
Enriched canonical pathways of mRNAs that are differentially expressed after iodide treatment (WTI/WTC, ≥1.5-fold change and *p* < 0.05) and are regulated by miRNAs that are also differentially expressed in the same samples. Analysis was performed using IPA with absolute *z*-score set at ≥1.5 and *p* < 0.05. White bars indicate upregulated pathways. Pathways marked with an asterisk (*) refer to processes relevant to inflammation/autoimmunity.

To get a more functional insight into the pairings of miRNAs–mRNAs from our dataset, pathway analysis was performed using as input the 542 mRNAs from our dataset that are targeted by differentially expressed miRNAs. This analysis highlighted pathways that are mostly related to inflammatory–immune responses such as Glycoprotein VI platelet (Gp6) signaling, Fcγ receptor phagocytosis, and PKCθ signaling in T cells (Figure 9).

### 3.6. The Transcriptomic Signature of the Murine Thyroid in Response to Excess Iodide Overlaps Significantly with the Signatures of Graves’ Disease in a Murine Model and of Human Papillary Thyroid Carcinoma (PTC)

**Figure 10:**
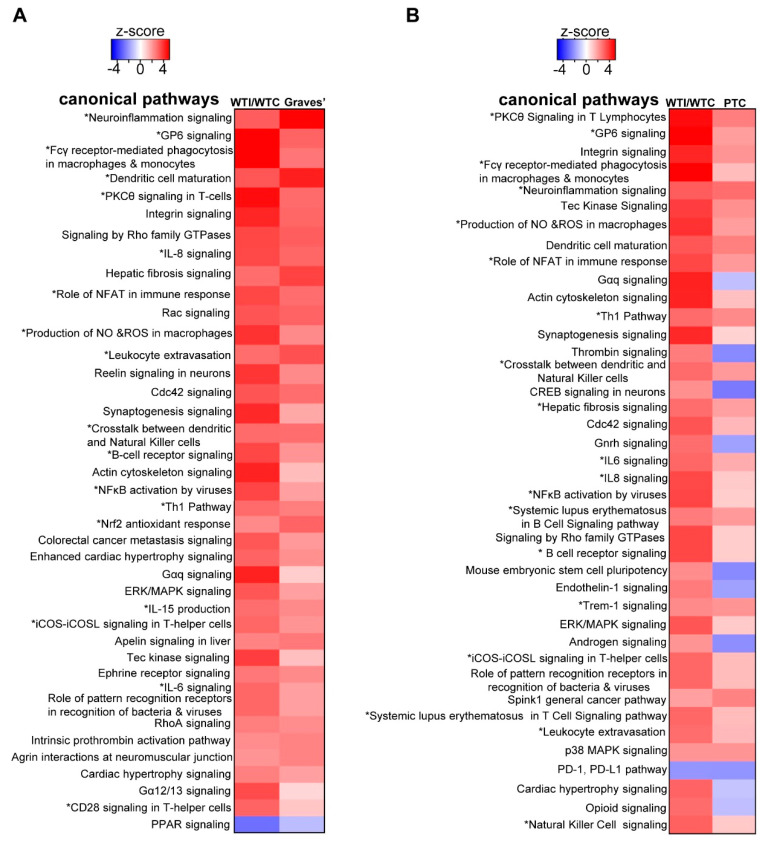
Comparison analyses using IPA of the top 40 enriched canonical pathways (*z*-score ≥2 and *p* < 0.05) in the thyroid in different pathophysiological settings. (**A**) Comparison of WT iodide-treated vs. non-treated mice (WTI/WT) and a genetic mouse model of Graves’ disease vs. respective WT controls (publicly available at Gene Expression Omnibus GSM955426-GSM955427). (**B**) Comparison of WT iodide-treated vs. non-treated mice (WTI/WT) and of human papillary thyroid carcinoma (PTC) vs. non-cancerous thyroid tissue from the same patient (publicly available at Gene Expression Omnibus GSM77362-GSM77379).

Lastly, we investigated whether the pathways enriched in response to iodide excess were also enriched in other settings of thyroid pathophysiology. To address this question, the pathway analysis profile of WT mice exposed to iodide was compared with publicly available datasets on thyroid samples using the “analysis match” function of the IPA platform. The results showed that the pathway enrichment profile of WT mice after iodide matched significantly with that of a murine model of Graves’ disease (thyroid autoimmune disease associated with hyperthyroidism) (overall average *z*-score match = 39.69%) that overexpresses CD40 in the thyroid [34]. Most of the overlapping pathways were related to oxidative stress (including the Nrf2 antioxidant response) and inflammation (marked with an asterisk (*) in Figure 10A), and the most highly enriched upstream regulators included TGFβ1, TNF, and NF-κB (Figure S2A, Supplementary Materials), among other inflammation- and fibrosis-related factors. The overlap of the canonical pathway signature between Nrf2 KO mice after iodide and the Graves’ disease model was even more extensive (overall average *z*-score match = 65.75%, Figure S3, Supplementary Materials).

The other pathway signature significantly overlapping that of iodide excess was derived from PTC compared to noncancerous thyroid tissue [35]. In this case also, several inflammation-related pathways were enriched in both conditions (marked with an asterisk (*) in Figure 10B). The most highly enriched upstream regulators (TNF, CSF2, and IFNG) were also related to inflammatory pathways (Figure S2B, Supplementary Materials).

## 4. Discussion

To the best of our knowledge, this is the first study that employed RNA-seq analysis to characterize the transcriptomic response of the thyroid to excess iodide. Moreover, the inclusion of Nrf2 KO mice in this experiment gave new insight into the roles of Nrf2 in thyroid economy. Specifically, under normal conditions (mice not treated with iodide), Nrf2 appears to regulate the expression of cytoprotective and antioxidant genes as is highlighted by the downregulation of the relevant pathways in the thyroids of Nrf2 KO mice (Figure 4A) and the lower expression levels of the Nrf2 target genes *Nqo1* and *Gpx2* (Figure 3B). This cytoprotective role of Nrf2 has been well documented in several other tissues, such as the liver [36]; by using Nrf2 loss- and gain-of-function mouse models, we recently showed that regulation of antioxidant genes by Nrf2 also occurs in the thyroid [2,37]. In these studies, roles of Nrf2 in thyroid economy beyond cytoprotection were also investigated, showing that Nrf2 is a positive regulator of *Tg* transcription and Tg protein abundance [2], as well as a negative regulator of Tg iodination [2]. We also showed that decreased expression of *Keap1*, which is associated with constitutive activation of Nrf2 signaling, can cause goiter with a tendency for hypothyroidism [37]. Identification of the full spectrum of Nrf2-regulated genes in the thyroid is important as a basis to further elucidate the mechanism underlying the aforementioned observations. Table S3 (Supplementary Materials) lists examples of potential Nrf2 target genes in the thyroid (genes that are downregulated in Nrf2 KO mice), some of which have shown similar expression patterns in other tissues as assessed from data found in publicly available databases. Several genes are already well-characterized Nrf2 targets (e.g., *Nqo1*, *Gpx2*), while others require further characterization. Last but not least, iodide exposure also activated Nrf2 signaling per se, as was evident by the upregulation of Nrf2 target genes (Figure 6B), in accordance with our previous in vitro and in vivo experiments [2] and the findings of others [9].

Iodide exposure elicited a distinct transcriptional response in thyroid that comprised pathways related to inflammation, (auto)immunity, fibrosis, and oxidative stress (Figure 1C and Figure 4B). A previous in vitro study with iodide-exposed human thyroid follicles [19] also described the induction of inflammation pathways and the thyroidal production of chemokines, such as C-C motif chemokine ligand 2 (CCL2) and C-X-C motif chemokine ligand 8 (CXCL8). However, this study was performed on thyroid samples from patients with Graves’ disease and, thus, their physiological relevance for normal thyroid tissue remains uncertain [19]. In the present study, we found that the iodide-induced mRNA changes are similar to the transcriptional changes observed in a genetic mouse model of Graves’ disease (Figure 10A); of note, that model showed upregulated Nrf2 signaling along with enhanced inflammation, fibrosis, and autoimmunity pathways [34]. We also found that lack of *Nrf2* augmented the inflammatory/(auto)immune response of thyroid to iodide (Figure 5 and Figure 6A). The tendencies of Nrf2 KO mice to develop multisystem autoimmunity with aging [38] and to show augmented inflammatory–innate immune response in models of sepsis [39] were already described and are in agreement with our present findings. Interestingly, we also found that the iodide-induced mRNA changes are similar to the transcriptional changes observed in human PTC samples (Figure 10B), which may be related to the fact that Nrf2 signaling is commonly activated in PTC as previously reported by our group [10] and others [12].

Another novelty of our findings lies in the description of Nrf2 as a modulator of the iodide-induced inflammatory response of the thyroid. Inflammatory cytokines (TNFα, IL6), NF-κB, and TGFβ signaling were predicted to be among the highly enriched upstream regulators of the iodide-induced transcriptional response, confirming the involvement of inflammation- and fibrosis-related pathways in this response (Figure 7A). Interestingly, *Tgfb* mRNA induction after iodide exposure was first described in sheep thyroid cells [40] and was confirmed in cultured human thyroid follicles from patients with Grave’s disease [17]. In our study, TGFβ and its fibrosis-related target genes were upregulated after iodide exposure, and this response was accentuated in the absence of Nrf2 (Figure 7A,B). Loss of Nrf2 function was shown to increase hepatic fibrosis in mice fed a high-fat diet [41] and to abrogate the protection against fibrosis after treatment with an Nrf2 pathway activator in mice fed a high-fat high-fructose diet [42]. The role of TGFβ in the thyroid has been studied mainly in cell culture systems, where it was found to inhibit iodide uptake [43], proliferation, and differentiation [44]. It was suggested that TGFβ may be secreted by the thyroid, with a possible autocrine antiproliferative function and a role in goitrogenesis [45]. More research is warranted to fully assess the role of TGFβ in thyroid economy under normal conditions and during iodide excess, as well as its interplay with Nrf2.

Although the present study mainly focused on alterations of mRNA expression after iodide exposure and their relevant pathway analyses, some preliminary analyses were also performed on changes of small RNA species, specifically lncRNAs and miRNAs. LncRNAs have relatively recently attracted interest in relation to thyroid disease, especially in thyroid cancer [46,47]. We found that 100 lncRNAs showed differential expression (≥2-fold change) after iodide treatment (Figure 8B). After eliminating the ones that show very low expression in any of the samples, the remaining 47 lncRNAs are listed in Table S6 (Supplementary Materials). Very few of them have been described before, and their exact roles are unknown. Therefore, extensive work is warranted to validate these results, including functional studies of the role of each lncRNA. While speculations about the latter should not be made at this point, Table S6 (Supplementary Materials) lists genes that might potentially be regulated by neighboring lncRNAs. Some of these genes may be of particular interesting in the thyroid; for instance, *Arid2* was shown to be mutated in poorly differentiated and anaplastic thyroid cancer [48], and *Akirin 2* is emerging as a novel player in the NF-κΒ-driven immune/inflammatory response [49].

Compared with lncRNA, miRNAs have been much more thoroughly studied in the thyroid, and some of them have actually entered clinical practice as parts of diagnostic panels to distinguish benign from malignant thyroid nodules in cases of indeterminate fine-needle aspiration cytology results [50,51]. In the present study, 70 miRNAs were found to respond to iodide excess (Tables S7 and S8, Supplementary Materials). By taking advantage of the study design, wherein sequencing for both mRNAs and miRNAs was performed in the same samples, the differentially expressed miRNAs were matched with their known or predicted mRNA targets that showed compatible changes in our dataset (i.e., increased miRNA expression decreased mRNA target expression, and vice versa). This pairing can form the basis for further studies on potential miRNA–mRNA iodide-regulated networks in thyroid. Noteworthy iodide-induced changes in thyroidal miRNAs include the downregulation of miR-204-5p and miR-218-5p and the upregulation of miR-128-3p, which all act as tumor suppressors in PTC [52,53,54]. Hence, the list of miRNAs whose thyroidal expression levels change in response to iodide excess can serve as a resource for further validation and analysis of their function in the setting of thyroid physiology and thyroid cancer.

Interestingly, the matched mRNA targets of differentially expressed miRNAs after iodide exposure outline a transcriptional program enriched in inflammation–autoimmunity and fibrosis pathways (Figure 9), which is essentially similar to what was found with using all the differentially expressed mRNAs as input (Figure 4B). Thus, it appears that this transcriptional response is integral to the physiological response of thyroid to excess iodide, and it may be important for thyroid economy, including thyroid autoregulation. In the absence of Nrf2, this inflammation–autoimmunity–fibrosis transcriptional program was accentuated (Figure 5, Figure 6 and Figure 7). Given that the Nrf2 pathway was activated in response to excess iodide [2,9] (Figure 6B), enhanced Nrf2 signaling is central to the thyroid’s defense program aiming to keep ROS and inflammation at bay.

The present study has several strengths and original aspects as compared to previous ones [17,18,19,21,55]: (i) this model examined the transcriptomic response of the thyroid to iodide in vivo; (ii) the method used to perform the transcriptomic profiling was next-generation sequencing; (iii) sequencing of both mRNAs and miRNAs in the same samples facilitated a matched analysis that highlighted networks of gene regulation in response to iodide; (iv) comparison of the data to transcriptomic profiles associated with Graves’ disease and PTC demonstrates a common involvement of inflammatory, immune, and fibrosis pathways; (v) in addition to WT mice, Nrf2 KO mice were included, yielding new insights into the roles of Nrf2 in thyroid economy, including suppression of inflammatory, immune and fibrosis pathways in response to iodide. However, the present study has some limitations. Firstly, as in other mouse thyroid studies, the very small size of the gland (~1 mg per lobe) makes it inevitable to dissect some or all of the (much smaller) parathyroid glands together with the thyroid and, thus, samples also contain parathyroid tissue. By applying a relatively high cutoff to exclude genes with low expression levels from downstream analyses, most parathyroid-specific genes were likely excluded, but some of them still appear in our analyses, such as the gene encoding parathyroid hormone (*Pth*) (Figure 2D); indeed, by chance, some samples could contain more parathyroid tissue than others. Nevertheless, interference with pathway analyses was apparently inconsiderable, not only because most of these genes should have been eliminated due to their low expression levels in the total sample, but also because pathway analyses studies take into account expression changes of groups of genes, rather than individual genes. Further research could focus on RNA-seq using microdissection of thyroid samples to ensure that no parathyroid tissue is present, or by employing techniques such as single-cell sequencing or deconvolution approaches to focus on gene expression changes in specific cell types. Secondly, it is noteworthy that *Tg* was absent among the DEGs, even though we previously showed that it is transcriptionally regulated by Nrf2, with lower expression levels in Nrf2 KO mice [2]. Exclusion of *Tg* from the gene lists was due to the Cufflinks suite algorithm that eliminates genes with unusually high expression levels.

## 5. Conclusions

In conclusion, excess iodide induces a distinct transcriptional response in the thyroid in vivo that favors inflammatory–autoimmune–fibrosis pathways along with enhanced Nrf2 signaling. Nrf2 activation by excess iodide attenuates the inflammatory–autoimmune–fibrosis response, which can have relevance for common thyroid pathologies such as Graves’ disease and PTC. Hence, synthetic or naturally occurring Nrf2 pathway modulators might be useful as preventive or supplemental therapeutic interventions in the setting of thyroid disease.

## Supplementary Materials

The following are available online at https://www.mdpi.com/2076-3921/9/9/884/s1.
