# Supplementary material for: The Transcriptomic Response of the Murine Thyroid Gland to Iodide Overload and the Role of the Nrf2 Antioxidant System
Source: Antioxidants (Basel). 2020 Sep 18;9(9):884. doi: 10.3390/antiox9090884 (PMC7555824; doi:10.3390/antiox9090884)
Supplement: Supplementary file 1 [file antioxidants-09-00884-s001.zip › Supp Figures 31.8.pdf]

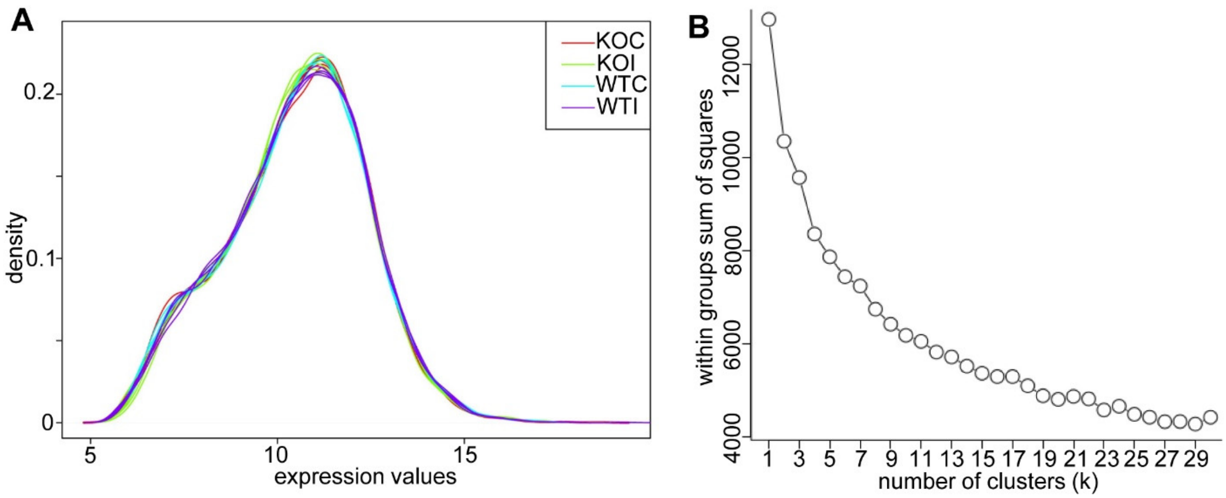

Supplemental Figure S1. A. Density plot of transformed counts data by EdgeR. To remove genes with very low expression, CPM (counts per million reads mapped) was set to 1 for all samples (n=16). **B.** "Elbow method" graph used for the k-means clustering (Fig.1C). The cutoff was set to 4 clusters, because adding further clusters did not reduce substantially the within-groups sum of squares. **C.** Heatmap and clustering of differentially expressed lncRNAs based on standardized expression values **D.** Heatmap and clustering of differentially expressed miRNAs based on standardized expression values. Samples are in rows and lncRNAs or miRNAs are in columns. **E.** Enriched canonical pathways of mRNAs that are differentially expressed after iodide treatment (WTI/WTC, fold change ≥1.5 and $p < 0.05$) and are regulated by miRNAs that are differentially expressed in the same samples. Analysis was performed using IPA with absolute z-score ≥1.5 and $p < 0.05$.

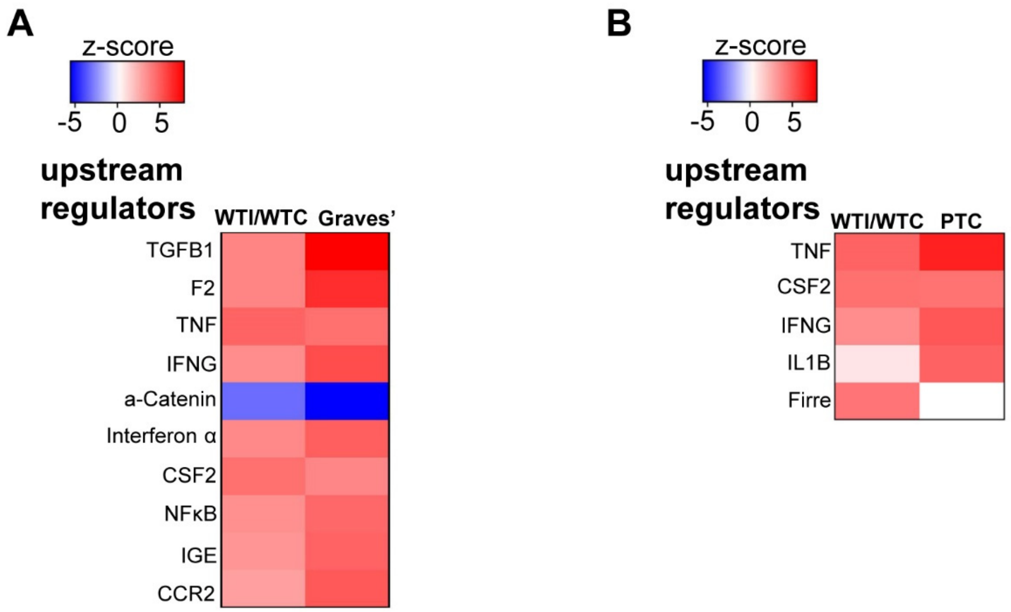

Supplemental Figure S2. Comparison analysis using IPA of the upstream regulators (z-score≥4 and p<0.01) in the thyroid in different pathophysiological settings. **A.** Comparison between WT iodide-treated vs. non-treated mice (WTI/WT) and a genetic mouse model of Graves' disease vs. respective WT controls (publicly available at Gene Expression Omnibus GSM955426-GSM955427). **B.** Comparison between WT iodide-treated vs. non-treated mice (WTI/WT) and human PTC vs. non-cancerous thyroid tissue from the same patient (publicly available at Gene Expression Omnibus GSM77362-GSM77379).

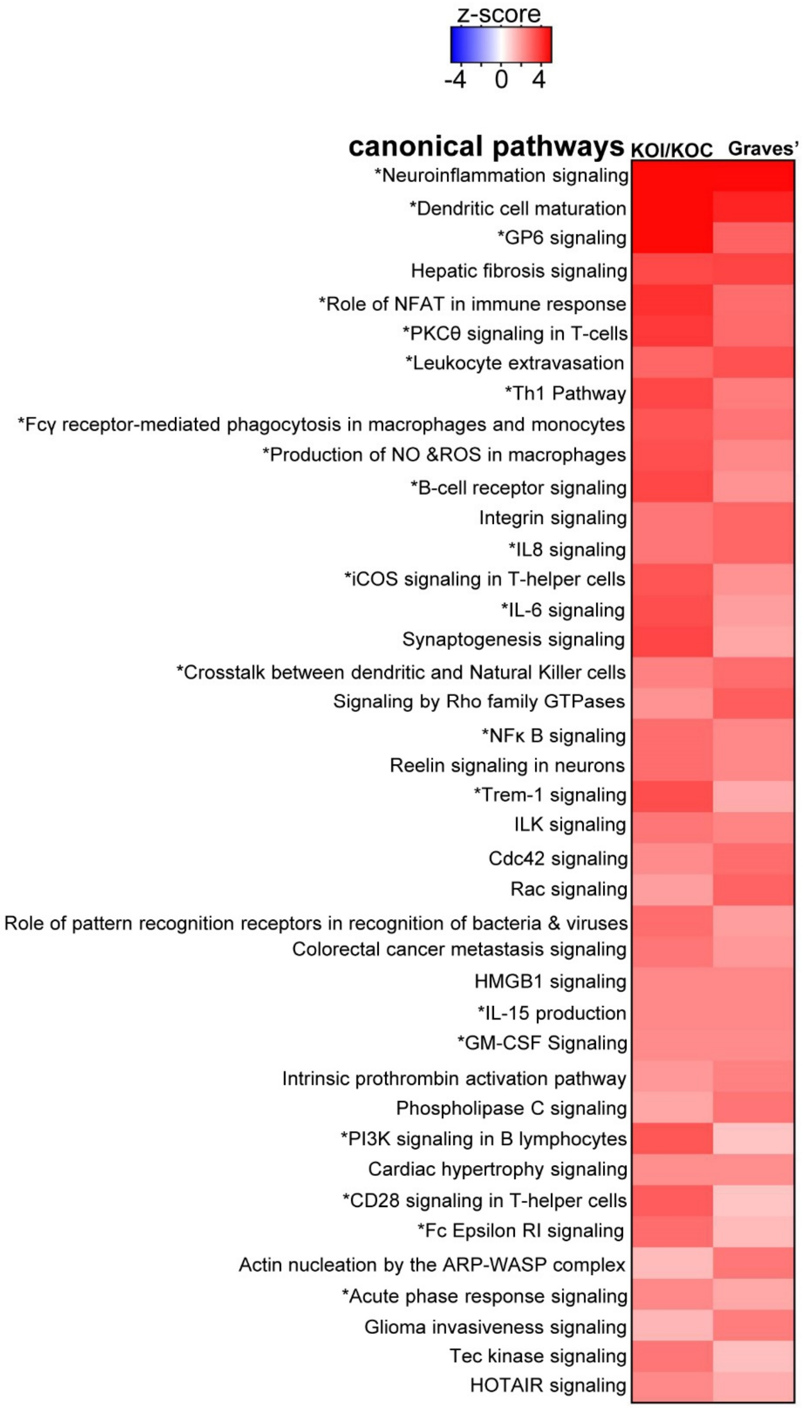

Supplemental Figure S3. Comparison analyses using IPA of the top 40 enriched canonical pathways (z-score ≥2 and p <0.05) in the thyroid between WT iodide-treated vs. non-treated Nrf2 knockout mice (KOI/KOC) and a genetic mouse model of Graves' disease vs. respective WT controls (publicly available at Gene Expression Omnibus GSM955426-GSM955427).
