# Supplementary material for: The Transcriptomic Response of the Murine Thyroid Gland to Iodide Overload and the Role of the Nrf2 Antioxidant System
Source: Antioxidants (Basel). 2020 Sep 18;9(9):884. doi: 10.3390/antiox9090884 (PMC7555824; doi:10.3390/antiox9090884)
Supplement: Supplementary file 1 [file antioxidants-09-00884-s001.zip › tables.zip!/tables/Table S3 genotype downregulated genes and compatible examples.docx]

**Table S2** Down-regulated genes in the thyroids of Nrf2KO mice.

| **Gene** | **Fold change** | **p-value** | **Datasets that show downregulation of each gene in loss of Nrf2 function studies** | **Datasets that show upregulation of each gene in gain of Nrf2 function studies** |
| --- | --- | --- | --- | --- |
| *Aox1* | -3.3 | 1.33E-32 | Lung (GSE18344, GSE58654, GSE9208,GSE29632), liver (GSE8969, GSE867) | N/A |
| *Nqo1* | -3.78 | 8.6E-31 | Liver (GSE85222), lung (GSE18344, GSE58654, GSE495, GSE9208, GSE29632), esophagus (GSE39629), | Liver (GSE80956), breast cells (GSE28813) |
| *Gpx2* | -10.2 | 7.17E-26 | Lung (GSE18344, GSE58654, GSE9208, GSE495, GSE29632), | Liver (GSE11287, GSE15633) |
| *Olfm4* | -4.51 | 2.87E-27 | N/A | N/A |
| *Gstm1* | -2.79 | 3.1E-18 | Lung (GSE18344, GSE9208, GSE58654, GSE29632 ), liver (GSE8969, GSE867,), esophagus (GSE39629), kidney (GSE48879), | Liver (GSE80956), breast cells (GSE28813) |
| *Cpeb1* | -2.16 | 2.38E-19 | N/A | N/A |
| *Wisp1* | -6.57 | 1.51E-13 | N/A | N/A |
| *Agr2* | -4.27 | 9.04E-12 | Lung (GSE29632) | N/A |
| *Gstm2* | -2.18 | 2.83E-10 | N/A | Liver (GSE80956) |
| *Gsta3* | -2.27 | 6.81E-10 | Lung (GSE29632, GSE18344, GSE9208, GSE495, GSE58654), esophagus (GSE39629), liver (GSE8969), kidney (GSE48879), | Colon (GSE68156) |
| *Map3k13* | -2.93 | 6.01E-08 | N/A | N/A |
| *Errfi1* | -7.74 | 1.59E-08 | N/A | N/A |
| *Gadd45a* | -2.48 | 1.498E-08 | N/A | N/A |
| *Pir* | -2.79 | 6.54E-08 | Lung (GSE58654, GSE18344), esophagus (GSE39629), | Liver (GSE80956), breast cells (GSE28813) |
| *Sgk1* | -3.36 | 0.000007 | N/A | N/A |
| *Sik1* | -3 | 0.000009 | N/A | N/A |
| *Ces1f* | -3.11 | 0.000010 | Lung (GSE18344, GSE58654, GSE9208), liver (GSE8969), esophagus (GSE39629) | N/A |
| *Derl3* | -4.09 | 0.000019 | N/A | Liver (GSE80956) |
| *Elf5* | -2.5 | 0.000003 | Lung (GSE18344, GSE9208, GSE495) | N/A |
| *Il15* | -2.16 | 0.000034 | N/A | N/A |
| *Fam46a* | -2.55 | 0.000025 | N/A | N/A |
| *Cebpd* | -11.82 | 0.00008 | N/A | N/A |
| *Ppp1r3c* | -2.11 | 0.000027 | N/A | N/A |
| *Slc17a9* | -2.6 | 0.00004 | N/A | N/A |
| *Slc14a2* | -5.62 | 0.00017 | N/A | N/A |
| *Pon1* | -2.87 | 0.00026 | Lung (GSE18344) | N/A |
| *Lgals12* | -2.1 | 0.00023 | N/A | N/A |
| *Pim3* | -2.9 | 0.0004 | N/A | N/A |
| *C7* | -2.01 | 0.00031 | N/A | N/A |
| *Cdh19* | -2.11 | 0.0002 | N/A | N/A |
| *Arl4d* | -3.43 | 0.0008 | N/A | N/A |
| *Fa2h* | -2.48 | 0.00078 | N/A | N/A |
| *Mettl21b* | -1.98 | 0.0043 | N/A | N/A |
| *Irf4* | -2.29 | 0.0012 | N/A | N/A |
| *Nrg4* | -1.75 | 0.0017 | N/A | N/A |

The fold-change for the expression of each gene in Nrf2KO versus WT mice is shown along with the p-value for this change and publicly available datasets (RNA-Seq, microarrays) that show compatible expression changes with our experimental observations. Genes with a p<0.01 and at least 2-fold change are shown and ranked by p-value. Genes that showed 0 expression in at least one of the samples were eliminated from this table. The datasets were discovered by using the “Knockdown Atlas” function of BaseSpace Correlation Engine (Illumina Inc). The prefix GSE refers to GEO DataSets (NCBI, NIH) and the datasets per gene are grouped depending on the tissue they refer to. Gain of Nrf2 function means Keap1 knockdown or Nrf2 overexpression and loss of Nrf2 function means Nrf2 knockout or Nrf2 knockdown). In the p-values column, “E-xx” means “10^-xx^”.
