# Supplementary material for: The Transcriptomic Response of the Murine Thyroid Gland to Iodide Overload and the Role of the Nrf2 Antioxidant System
Source: Antioxidants (Basel). 2020 Sep 18;9(9):884. doi: 10.3390/antiox9090884 (PMC7555824; doi:10.3390/antiox9090884)
Supplement: Supplementary file 1 [file antioxidants-09-00884-s001.zip › tables.zip!/tables/Table S7 miRNA treatment UP.docx]

**Table 5. miRNAs that are upregulated in the thyroid after iodide treatment**

| **miRNAs** | **Fold**  **change** | **p-value** |
| --- | --- | --- |
| mmu-miR-133a-3p | 11.61 | 0.00438 |
| mmu-miR-669c-5p | 5.28 | 0.00003 |
| mmu-miR-6956-3p | 4.5 | 0.0004 |
| mmu-miR-505-5p | 3.65 | 0.00064 |
| mmu-miR-344-3p | 3.56 | 0.00004 |
| mmu-miR-503-5p | 3.13 | 0.00682 |
| mmu-miR-7015-3p | 2.68 | 0.00667 |
| mmu-miR-504-5p | 2.56 | 0.0384 |
| mmu-miR-129-1-3p | 2.41 | 0.03555 |
| mmu-miR-92b-3p | 2.39 | 0.00553 |
| mmu-miR-1306-3p | 2.27 | 0.01599 |
| mmu-miR-149-5p | 2.24 | 0.00449 |
| mmu-miR-34c-5p | 2.23 | 0.00707 |
| mmu-miR-3074-5p | 2.22 | 0.01618 |
| mmu-miR-7043-3p | 2.21 | 0.00429 |
| mmu-miR-1306-5p | 2.11 | 0.0299 |
| mmu-miR-484 | 2.02 | 0.00181 |
| mmu-miR-6899-3p | 1.99 | 0.02721 |
| mmu-miR-676-3p | 1.94 | 0.01068 |
| mmu-miR-130b-5p | 1.92 | 0.00779 |
| mmu-miR-1964-3p | 1.89 | 0.03267 |
| mmu-miR-485-3p | 1.85 | 0.01186 |
| mmu-miR-423-5p | 1.84 | 0.03469 |
| mmu-miR-96-5p | 1.84 | 0.00158 |
| mmu-miR-128-3p | 1.82 | 0.00528 |
| mmu-miR-351-5p | 1.81 | 0.00423 |
| mmu-miR-6952-3p | 1.8 | 0.04042 |
| mmu-miR-326-3p | 1.79 | 0.01265 |
| mmu-miR-184-3p | 1.79 | 0.01856 |
| mmu-let-7d-3p | 1.74 | 0.03414 |
| mmu-miR-423-3p | 1.74 | 0.00996 |
| mmu-let-7b-3p | 1.74 | 0.02431 |
| mmu-miR-152-5p | 1.71 | 0.01627 |
| mmu-miR-142a-5p | 1.71 | 0.0157 |
| mmu-miR-25-5p | 1.69 | 0.03719 |
| mmu-let-7e-3p | 1.68 | 0.01644 |
| mmu-miR-330-3p | 1.61 | 0.01404 |
| mmu-let-7f-1-3p | 1.59 | 0.04791 |
| mmu-miR-98-5p | 1.56 | 0.02428 |
| mmu-miR-22-3p | 1.54 | 0.00764 |
| mmu-miR-700-3p | 1.52 | 0.04778 |
| mmu-miR-92a-3p | 1.5 | 0.04582 |
| mmu-miR-99b-3p | 1.41 | 0.043 |
| mmu-let-7d-5p | 1.4 | 0.03713 |

The fold-change of the expression of each miRNA after iodide treatment is shown along with the p-value.
