# Supplementary material for: The Transcriptomic Response of the Murine Thyroid Gland to Iodide Overload and the Role of the Nrf2 Antioxidant System
Source: Antioxidants (Basel). 2020 Sep 18;9(9):884. doi: 10.3390/antiox9090884 (PMC7555824; doi:10.3390/antiox9090884)
Supplement: Supplementary file 1 [file antioxidants-09-00884-s001.zip › tables.zip!/tables/Table S8 miRNA treatment down.docx]

**Table 6. miRNAs that are downregulated in the thyroid after iodide treatment**

| **miRNAs** | **Fold change** | **p-value** |
| --- | --- | --- |
| mmu-miR-29b-1-5p | -2.42 | 0.03778 |
| mmu-miR-712-5p | -2.41 | 0.00313 |
| mmu-miR-878-3p | -2.28 | 0.00884 |
| mmu-miR-194-5p | -2.11 | 0.00926 |
| mmu-miR-2137 | -2.08 | 0.0095 |
| mmu-miR-708-5p | -2.06 | 0.00174 |
| mmu-miR-743a-3p | -2.05 | 0.02093 |
| mmu-miR-135a-5p | -1.97 | 0.00118 |
| mmu-miR-1195 | -1.93 | 0.0144 |
| mmu-miR-5126 | -1.88 | 0.0453 |
| mmu-miR-193a-5p | -1.85 | 0.01993 |
| mmu-miR-135a-2-3p | -1.82 | 0.00421 |
| mmu-miR-3470a | -1.81 | 0.00618 |
| mmu-miR-195a-5p | -1.77 | 0.04904 |
| mmu-miR-872-5p | -1.74 | 0.03264 |
| mmu-miR-880-3p | -1.69 | 0.01676 |
| mmu-miR-143-5p | -1.68 | 0.0066 |
| mmu-miR-27b-5p | -1.68 | 0.00312 |
| mmu-miR-881-3p | -1.66 | 0.04827 |
| mmu-miR-141-3p | -1.61 | 0.03303 |
| mmu-miR-16-5p | -1.53 | 0.03807 |
| mmu-miR-126a-3p | -1.52 | 0.01921 |
| mmu-miR-101b-3p | -1.5 | 0.04825 |
| mmu-miR-9-5p | -1.5 | 0.02043 |
| mmu-miR-218-5p | -1.46 | 0.01463 |
| mmu-miR-204-5p | -1.39 | 0.04168 |

The fold-change of the expression of each miRNA after iodide treatment is shown along with the p-value.
