# Supplementary material for: The Transcriptomic Response of the Murine Thyroid Gland to Iodide Overload and the Role of the Nrf2 Antioxidant System
Source: Antioxidants (Basel). 2020 Sep 18;9(9):884. doi: 10.3390/antiox9090884 (PMC7555824; doi:10.3390/antiox9090884)
Supplement: Supplementary file 1 [file antioxidants-09-00884-s001.zip › tables.zip!/tables/Table S9 miRNA-mRNA pairs.docx]

**Table 3. Differentially expressed miRNAs along with their differentially expressed target mRNAs after iodide treatment in WT mice (WTI/WTC)**

| miRNA | miRNA target genes |
| --- | --- |
| miR-195a-5p | Flt3, Arhgdia, Tmem43, Ucp2, Mapk3, Zyx, Atg9a, Serpine2, Kif23, Lypla2, Cdk6 |
| miR-204-5p | Erf, Arpc1b, Itgb4, Cdh11 |
| miR-218-5p | Col1a1, Lamb3 |
| miR-135a-5p | Alox5ap |
| miR-351-5p | Acss1, Igfbp3 |
| miR-128-3p | Dbi, Ntrk3, Snap25 |

Blue and red font indicate downregulation and upregulation respectively. Herein, only interactions of high confidence are depicted (they have been experimentally observed in the bibliography curated by IPA). The differentially expressed miRNAs were paired with the differentially expressed mRNAs from the same samples following the rule of opposite regulation; increased miRNA after treatment matched with decreased mRNA target and decreased miRNA matched with increased mRNA target. microRNA target filter analysis was used in IPA. **Table S6** includes all miRNA-mRNA pairs.
